# Depression, anxiety, substance misuse and self-harm in children and young people with rare chronic liver disease

**DOI:** 10.1192/bjo.2022.550

**Published:** 2022-07-28

**Authors:** Wai Hoong Chang, Graham R. Foster, Deirdre A. Kelly, Alvina G. Lai

**Affiliations:** Institute of Health Informatics, University College London, UK; Barts Liver Centre, Blizard Institute, Queen Mary University of London, UK; Liver Unit, Birmingham Women's & Children's Hospital, Birmingham, UK; and Institute of Immunology and Immunotherapy, University of Birmingham, UK

**Keywords:** Mental illness, anxiety, depression, self-harm, substance misuse

## Abstract

The burden of mental illness in young people with chronic liver disease is not known. In this population cohort study in England, we identified 358 individuals (aged ≤25 years) diagnosed with autoimmune hepatitis or liver disease related to cystic fibrosis and 1541 propensity-score-matched controls. By the first year of follow-up, the cumulative burden of psychiatric events in participants with liver disease was high compared with controls: anxiety disorder (6.87 per 100 individuals [95% CI 4.00–9.73] *v*. 2.22 [95% CI 1.37–3.07]), depression (5.10 [95% CI 2.83–7.37] *v*. 0.86 [95% CI 0.53–1.19]), substance misuse (10.61 [95% CI 9.50–11.73] *v*. 1.23 [95% CI 0.71–1.75]) and self-harm (3.09 [95% CI 1.12–5.05] *v*. 0.20 [95% CI 0.07–0.33]). Participants with liver disease had a 2-fold increase (OR = 1.94, 95% CI 1.45–2.58), a 2.5-fold increase (OR = 2.59, 95% CI 1.91–3.50) and 4.4-fold increase (OR = 4.44; 95% CI 3.46–5.71) in the risk of anxiety, depression and substance misuse, respectively. These findings highlight the need for effective intervention in psychiatric disorders in young people with rare liver disease.

Mental illness in children often goes unnoticed and untreated. Adolescents with paediatric-onset physical conditions have an even higher risk of developing mental illness;^1^ 1.7 million children and young people in England live with chronic conditions.^2^ This study aims to investigate the burden of anxiety, depression, substance misuse and self-harm in young people with chronic liver disease. We selected liver disease because it is often rare and severe in children. We also investigated self-harm as it is an important risk factor for premature mortality and further self-harm episodes.^3^ The National Institute for Health and Care Excellence (NICE) guideline highlights that young people with diabetes are at a high risk of depression and anxiety^4^ and the American Diabetes Association highlighted the importance of psychosocial care in their latest position statement.^5^ In contrast, research and guidelines on mental illness in children with rare conditions have remained scarce, in part owing to small sample sizes. This study therefore focuses on two rare chronic liver conditions (autoimmune hepatitis and liver disease related to cystic fibrosis). Although cystic fibrosis is a multi-organ genetic disease, liver disease is the second most common cause of mortality after respiratory disease, and people with cystic fibrosis and liver disease have three times higher risk of death compared with those without liver disease.^6^ The UK's public healthcare system presents a unique opportunity to employ population cohorts to inform the development of effective targeted policies for young people with long-term liver conditions.

## Method

We employed primary and secondary care data-sets for patients in England from 1998 to 2020. The data custodian is the Medicines and Healthcare products Regulatory Agency. Variables available for this study include diagnoses, date of diagnosis, gender, year of birth and deprivation status. The study participants were 1899 young people (358 with liver disease and 1541 controls). We used the mean cumulative count (MCC) method^7^ to capture the total burden of anxiety, depression, substance misuse and non-fatal self-harm in young people aged ≤25 years with autoimmune hepatitis or liver disease related to cystic fibrosis. We used open-access electronic health record phenotypes (https://phenotypes.healthdatagateway.org/home) for anxiety, depression, substance misuse and self-harm that were previously validated.^8,9^

Since patients might experience recurrent psychiatric events, the MCC method, unlike cumulative incidence, summarises all events that occurred and not just the first event. An event is defined as in-patient hospital admission or a primary care visit. MCC was used to estimate the average number of healthcare events encountered by young people with chronic liver disease compared with matched controls. We focused on anxiety and depression because they are the most common mental health conditions in young people. We did not include conditions that are likely to affect cognitive functioning (e.g. autism) because developmental delays or neurological problems might complicate the identification of mental health events. The burden of recurrent events was analysed with deaths considered as competing risk events.^7^ The 95% confidence intervals were generated using the bootstrap percentile method.^10^

Controls (young people without chronic liver disease) were identified using propensity score matching, where they are matched by year of birth, Index of Multiple Deprivation (a measure of relative deprivation and poverty) and gender. Matching was performed using the optimal pair matching algorithm in the R matchit package (using RStudio for MacOS) to keep the sum of the absolute pairwise distances in the matched sample as small as possible. Index dates (start of follow-up) for cases were the dates of liver disease diagnosis. Index dates for controls were assigned based on the index date of their corresponding matched cases. Individuals were followed up until the date of deregistration from the healthcare practice, date of administrative censoring (31 October 2020) or death, whichever occurred first.

We explored the presence of three additional conditions (asthma, diabetes and epilepsy) in the cohort. A second control group was created to exclude individuals with asthma, diabetes or epilepsy. Subgroup analyses on this second control group compared with individuals with liver disease were performed.

### Ethics approval

Information governance approval was obtained from the Medicines and Healthcare products Regulatory Agency (21_593). Consent to participate was not applicable.

## Results

We identified 358 patients aged ≤25 years who had a diagnosis of autoimmune hepatitis or liver disease related to cystic fibrosis (supplementary Table 1 available at https://doi.org/10.1192/bjo.2022.550). We used propensity score matching to identify 1541 controls. Mean follow-up times for patients and controls were 8.38 years (IQR = 9.79) and 11.33 years (IQR = 10.67) respectively.

**Fig. 1:**
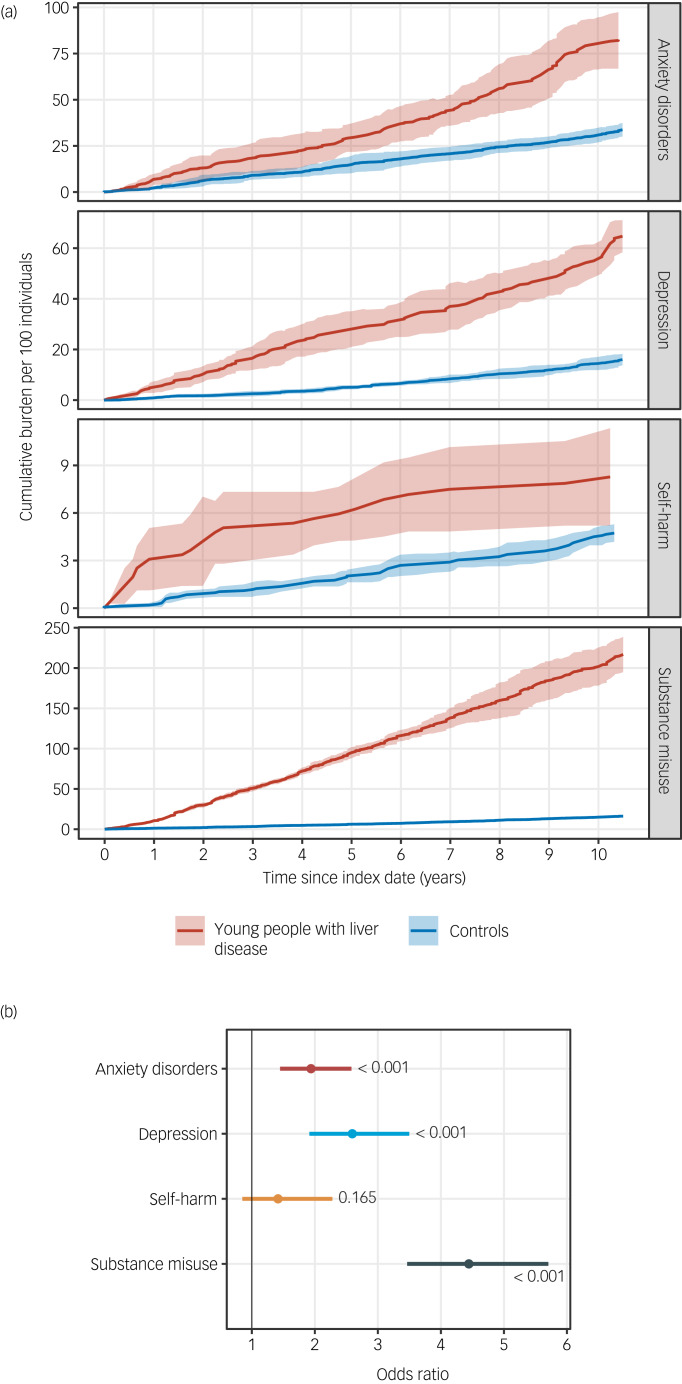
(a) Cumulative burden of anxiety, depression, substance misuse and self-harm in young people with chronic liver disease compared with controls. (b) Logistic regression analysis of the association between liver disease and mental illness: error bars show 95% confidence intervals, and *P*-values are shown on the plot.

We observed that young people with chronic liver disease had a higher burden of anxiety disorder, depression, substance misuse and self-harm compared with matched controls. By the first year of follow-up, the cumulative burdens in young people with liver disease compared with controls were as follow: anxiety disorder (6.87 per 100 individuals [95% CI 4.00–9.73] *v*. 2.22 [95% CI 1.37–3.07] in controls), depression (5.10 [95% CI 2.83–7.37] *v*. 0.86 [95% CI 0.53–1.19] in controls), substance misuse (10.61 [95% CI 9.50–11.73] *v*. 1.23 [95% CI 0.71–1.75] in controls) and non-fatal self-harm (3.09 [95% CI 1.12–5.05] *v*. 0.20 [95% CI 0.07–0.33] in controls) (Fig. 1, Supplementary Table 2).

By the fifth year of follow-up, the cumulative burden in young people with liver disease compared with controls had increased to: anxiety disorder (29.45 [95% CI 21.81–37.08] *v*. 14.84 [95% CI 10.17–19.50] in controls), depression (28.02 [95% CI 21.02–35.03] *v*. 5.01 [95% CI 4.16–5.86] in controls), substance misuse (95.04 [95% CI 88.18–101.90] *v*. 6.19 [95% CI 5.01–7.38] in controls) and self-harm (5.94 [95% CI 4.24–7.64] *v*. 1.96 [95% CI 1.57–2.36] in controls) (Fig. 1, supplementary Table 2).

Young people with liver disease had an almost 2-fold increase (odds ratio OR = 1.94; 95% CI 1.45–2.58; *P* < 0.001) in the risk of developing anxiety, a 2.5-fold (OR = 2.59; 95% CI 1.91–3.50; *P* < 0.001) increase in the risk of depression and 4.4-fold increase (OR = 4.44; 95% CI 3.46–5.71) in the risk of substance misuse (supplementary Table 3).

We performed subgroup analyses on another control group after excluding individuals with asthma, diabetes and epilepsy. Results appeared to be largely unaffected (supplementary Tables 2 and 3).

## Discussion

Our findings suggest a strong association between rare liver conditions and four mental disorders (anxiety, depression, substance misuse and self-harm) in young people. Healthcare systems are affected by understaffing and under-resourcing, and mental health services have taken a back seat, which has been exacerbated by the COVID-19 pandemic. Although things are slowly improving, the level of mental healthcare across communities and populations remains inequitable, with certain groups, such as young people, bearing the brunt of inadequate resourcing.

A systematic review of 137 studies found limited evidence that school-based interventions for preventing anxiety or depression were effective.^11^ This highlights the need for future interventions to address wider structural and familial contexts, particularly in children with additional physical challenges. Over 50% of children reported problems related to mental disorders after being diagnosed with a physical condition^12^ and a significant association has been found between the severity of physical conditions and mental disorders in young people.^13^

We acknowledge several limitations in this study. Controls were matched only on gender, deprivation and year of birth. We were unable to assess the effects of treatment for mental illness. We also do not have access to free-text medical notes or letters. We considered only liver disease related to cystic fibrosis but have not investigated the psychiatric burden for individuals with cystic fibrosis in the absence of liver disease.

Liver disease in children is often under-recognised, misunderstood and diagnosed late. Exacerbated by the gaps in mental health provision for young people, the long-term economic and social ramifications of untreated mental illness become a very dire prospect. These results underpin a call for action to better treat mental illness in young people, to open conversations between parents, doctors and young people by promoting awareness and overcoming stigma and to reinforce the capacity of mental health research by facilitating scaled data access and analytics to fill gaps in evidence.

## Data Availability

The data used in this study are available on successful ethics application to the Medicines and Healthcare products Regulatory Agency. All summarised data and results are made available in the online supplementary material.

## Supplementary material

For supplementary material accompanying this paper visit http://doi.org/10.1192/bjo.2022.550. click here to view supplementary material
