# Supplementary material for: Depression, anxiety, substance misuse and self-harm in children and young people with rare chronic liver disease
Source: BJPsych Open. 2022 Jul 28;8(5):e146. doi: 10.1192/bjo.2022.550 (PMC9380248; doi:10.1192/bjo.2022.550)
Supplement: Supplementary file 1 [file S2056472422005506sup001.pdf]

Table S1. Baseline characteristics

	Controls	Liver disease
	1541	358
**Male (%)**	861 (55.9)	168 (46.9)
**Age at cohort entry (mean [SD])**	16.90 (6.23)	17.89 (5.30)
**Index of Multiple Deprivation**		
Least deprived (IMD 1)	323 (21.0)	70 (19.6)
IMD2	283 (18.4)	69 (19.3)
IMD3	271 (17.6)	73 (20.4)
IMD4	340 (22.1)	85 (23.7)
Most deprived (IMD5)	321 (20.9)	61 (17.0)
**Presence of other conditions**		
Asthma (%)	255 (16.5)	103 (28.8)
Epilepsy (%)	8 (0.5)	2 (0.6)
Diabetes (%)	30 (1.9)	74 (20.7)
**Mental disorders**		
Anxiety disorders (%)	211 (13.7)	85 (23.7)
Depression (%)	156 (10.1)	83 (23.2)
Self-harm (%)	75 (4.9)	23 (6.4)
Substance abuse (%)	273 (17.7)	175 (48.9)

Table S2. Cumulative burden of events for anxiety disorders, depression, substance abuse and non-fatal self-harm in young people with chronic liver disease versus controls. Cumulative burden is shown as mean number of events per 100 individuals.

Groups	Outcome	Years of follow-up	Cumulative burden	Lower CI	Upper CI
**With liver disease**	Anxiety disorders	1	6.87	4.00	9.73
**With liver disease**	Anxiety disorders	2	12.61	5.72	19.50
**With liver disease**	Anxiety disorders	3	18.39	10.34	26.44
**With liver disease**	Anxiety disorders	4	22.52	13.32	31.73
**With liver disease**	Anxiety disorders	5	29.45	21.81	37.08
**With liver disease**	Anxiety disorders	6	36.99	27.50	46.48
**With liver disease**	Anxiety disorders	7	44.25	35.90	52.60
**With liver disease**	Anxiety disorders	8	56.03	42.85	69.21
**With liver disease**	Anxiety disorders	9	66.29	50.72	81.86
**With liver disease**	Anxiety disorders	10	80.47	66.38	94.56
**Controls**	Anxiety disorders	1	2.22	1.37	3.07
**Controls**	Anxiety disorders	2	6.20	3.07	9.33
**Controls**	Anxiety disorders	3	9.08	6.74	11.43
**Controls**	Anxiety disorders	4	10.79	8.18	13.41
**Controls**	Anxiety disorders	5	14.84	10.17	19.50
**Controls**	Anxiety disorders	6	17.93	13.80	22.06
**Controls**	Anxiety disorders	7	20.76	17.01	24.50
**Controls**	Anxiety disorders	8	24.39	21.20	27.58
**Controls**	Anxiety disorders	9	26.92	23.81	30.02
**Controls**	Anxiety disorders	10	30.86	26.93	34.79
**Controls (without asthma, diabetes or epilepsy)**	Anxiety disorders	1	2.08	1.20	2.96
**Controls (without asthma, diabetes or epilepsy)**	Anxiety disorders	2	6.80	4.88	8.72
**Controls (without asthma, diabetes or epilepsy)**	Anxiety disorders	3	9.93	7.78	12.09
**Controls (without asthma, diabetes or epilepsy)**	Anxiety disorders	4	11.47	9.40	13.53
**Controls (without asthma, diabetes or epilepsy)**	Anxiety disorders	5	14.06	11.19	16.92
**Controls (without asthma, diabetes or epilepsy)**	Anxiety disorders	6	16.84	15.93	17.76
**Controls (without asthma, diabetes or epilepsy)**	Anxiety disorders	7	19.86	18.75	20.97
**Controls (without asthma, diabetes or epilepsy)**	Anxiety disorders	8	23.25	21.18	25.33
**Controls (without asthma, diabetes or epilepsy)**	Anxiety disorders	9	26.06	23.20	28.92
**Controls (without asthma, diabetes or epilepsy)**	Anxiety disorders	10	29.82	26.79	32.86
**With liver disease**	Depression	1	5.10	2.83	7.37
**With liver disease**	Depression	2	10.21	7.10	13.32
**With liver disease**	Depression	3	16.51	11.93	21.10
**With liver disease**	Depression	4	23.54	17.17	29.91
**With liver disease**	Depression	5	28.02	21.02	35.03
**With liver disease**	Depression	6	31.75	24.95	38.55
**With liver disease**	Depression	7	36.98	27.85	46.12
**With liver disease**	Depression	8	42.75	35.50	50.00
**With liver disease**	Depression	9	48.12	41.29	54.94
**With liver disease**	Depression	10	55.79	49.24	62.34
**Controls**	Depression	1	0.86	0.53	1.19
**Controls**	Depression	2	1.72	0.99	2.45
**Controls**	Depression	3	2.52	1.46	3.59
**Controls**	Depression	4	3.46	2.60	4.32
**Controls**	Depression	5	5.01	4.16	5.86
**Controls**	Depression	6	6.58	5.75	7.40
**Controls**	Depression	7	8.39	6.73	10.06
**Controls**	Depression	8	10.38	8.34	12.41
**Controls**	Depression	9	11.91	9.88	13.95
**Controls**	Depression	10	14.44	11.85	17.03
**Controls (without asthma, diabetes or epilepsy)**	Depression	1	0.98	0.57	1.38
**Controls (without asthma, diabetes or epilepsy)**	Depression	2	1.95	1.14	2.76
**Controls (without asthma, diabetes or epilepsy)**	Depression	3	2.60	1.87	3.34
**Controls (without asthma, diabetes or epilepsy)**	Depression	4	3.75	2.44	5.06
**Controls (without asthma, diabetes or epilepsy)**	Depression	5	5.39	3.68	7.11
**Controls (without asthma, diabetes or epilepsy)**	Depression	6	7.21	5.48	8.94
**Controls (without asthma, diabetes or epilepsy)**	Depression	7	8.57	6.25	10.89
**Controls (without asthma, diabetes or epilepsy)**	Depression	8	10.63	8.28	12.98
**Controls (without asthma, diabetes or epilepsy)**	Depression	9	12.22	9.64	14.79
**Controls (without asthma, diabetes or epilepsy)**	Depression	10	14.53	11.67	17.39
**With liver disease**	Self-harm	1	3.09	1.12	5.05
**With liver disease**	Self-harm	2	4.21	1.40	7.02
**With liver disease**	Self-harm	3	5.06	2.80	7.33
**With liver disease**	Self-harm	4	5.35	3.38	7.33
**With liver disease**	Self-harm	5	5.94	4.24	7.64
**With liver disease**	Self-harm	6	6.85	4.51	9.19
**With liver disease**	Self-harm	7	7.49	4.84	10.15
**With liver disease**	Self-harm	8	7.49	4.84	10.15
**With liver disease**	Self-harm	9	7.49	4.84	10.15
**With liver disease**	Self-harm	10	7.87	5.20	10.53
**Controls**	Self-harm	1	0.20	0.07	0.33
**Controls**	Self-harm	2	0.85	0.59	1.11
**Controls**	Self-harm	3	1.17	0.72	1.63
**Controls**	Self-harm	4	1.56	1.24	1.89
**Controls**	Self-harm	5	1.96	1.57	2.36
**Controls**	Self-harm	6	2.70	2.03	3.37
**Controls**	Self-harm	7	2.90	2.30	3.51
**Controls**	Self-harm	8	3.25	2.64	3.86
**Controls**	Self-harm	9	3.60	2.93	4.28
**Controls**	Self-harm	10	4.50	3.96	5.04
**Controls (without asthma, diabetes or epilepsy)**	Self-harm	1	0.24	0.08	0.40
**Controls (without asthma, diabetes or epilepsy)**	Self-harm	2	1.04	0.40	1.67
**Controls (without asthma, diabetes or epilepsy)**	Self-harm	3	1.43	0.72	2.15
**Controls (without asthma, diabetes or epilepsy)**	Self-harm	4	1.75	1.04	2.47
**Controls (without asthma, diabetes or epilepsy)**	Self-harm	5	2.16	1.52	2.79
**Controls (without asthma, diabetes or epilepsy)**	Self-harm	6	2.89	2.01	3.77
**Controls (without asthma, diabetes or epilepsy)**	Self-harm	7	3.05	2.17	3.94
**Controls (without asthma, diabetes or epilepsy)**	Self-harm	8	3.30	2.25	4.35
**Controls (without asthma, diabetes or epilepsy)**	Self-harm	9	3.65	2.51	4.79
**Controls (without asthma, diabetes or epilepsy)**	Self-harm	10	4.64	3.57	5.72
**With liver disease**	Substance abuse	1	10.61	9.50	11.73
**With liver disease**	Substance abuse	2	29.93	26.84	33.02
**With liver disease**	Substance abuse	3	50.82	47.07	54.58
**With liver disease**	Substance abuse	4	72.15	67.90	76.40
**With liver disease**	Substance abuse	5	95.04	88.18	101.90
**With liver disease**	Substance abuse	6	116.18	109.30	123.07
**With liver disease**	Substance abuse	7	138.16	125.52	150.80
**With liver disease**	Substance abuse	8	159.86	137.65	182.08
**With liver disease**	Substance abuse	9	184.59	160.82	208.35
**With liver disease**	Substance abuse	10	201.82	178.22	225.42
**Controls**	Substance abuse	1	1.23	0.71	1.75
**Controls**	Substance abuse	2	2.08	1.23	2.92
**Controls**	Substance abuse	3	3.31	2.27	4.35
**Controls**	Substance abuse	4	4.75	3.90	5.60
**Controls**	Substance abuse	5	6.19	5.01	7.38
**Controls**	Substance abuse	6	7.33	5.94	8.71
**Controls**	Substance abuse	7	9.23	7.86	10.60
**Controls**	Substance abuse	8	11.16	10.06	12.25
**Controls**	Substance abuse	9	13.07	11.36	14.78
**Controls**	Substance abuse	10	14.99	13.31	16.68
**Controls (without asthma, diabetes or epilepsy)**	Substance abuse	1	0.80	0.64	0.95
**Controls (without asthma, diabetes or epilepsy)**	Substance abuse	2	1.59	1.19	1.99
**Controls (without asthma, diabetes or epilepsy)**	Substance abuse	3	2.87	1.91	3.82
**Controls (without asthma, diabetes or epilepsy)**	Substance abuse	4	3.98	3.27	4.70
**Controls (without asthma, diabetes or epilepsy)**	Substance abuse	5	5.03	4.15	5.90
**Controls (without asthma, diabetes or epilepsy)**	Substance abuse	6	6.24	5.44	7.04
**Controls (without asthma, diabetes or epilepsy)**	Substance abuse	7	8.14	7.61	8.67
**Controls (without asthma, diabetes or epilepsy)**	Substance abuse	8	9.99	8.91	11.07
**Controls (without asthma, diabetes or epilepsy)**	Substance abuse	9	11.88	10.62	13.13
**Controls (without asthma, diabetes or epilepsy)**	Substance abuse	10	13.94	11.96	15.92

Table S3. Logistic regression analysis for the risk of anxiety disorder, depression, substance abuse and non-fatal self-harm. Case (young people with chronic liver disease) and control groups were obtained via propensity score matching. Controls were matched by year of birth, Index of Multiple Deprivation and sex.

Outcome	Odds ratio	Lower CI	Upper CI	P value	Analysis
**Depression**	2.59	1.91	3.50	< 0.001	Controls vs. cases
**Anxiety disorders**	1.94	1.45	2.58	< 0.001	Controls vs. cases
**Self-harm**	1.42	0.85	2.28	0.165	Controls vs. cases
**Substance abuse**	4.44	3.46	5.71	< 0.001	Controls vs. cases
**Depression**	2.79	2.03	3.81	< 0.001	Controls (without asthma, diabetes and epilepsy) vs. cases
**Anxiety disorders**	2.26	1.67	3.05	< 0.001	Controls (without asthma, diabetes and epilepsy) vs. cases
**Self_harm**	1.41	0.84	2.30	0.184	Controls (without asthma, diabetes and epilepsy) vs. cases
**Substance abuse**	4.68	3.62	6.07	< 0.001	Controls (without asthma, diabetes and epilepsy) vs. cases
